# Physiological implications of life at the forest interface of oil palm agriculture: blood profiles of wild Malay civets (*Viverra tangalunga*)

**DOI:** 10.1093/conphys/coaa127

**Published:** 2020-12-30

**Authors:** Meaghan N Evans, Sergio Guerrero-Sanchez, Peter Kille, Carsten T Müller, Mohd Soffian Abu Bakar, Benoit Goossens

**Affiliations:** 1 School of Biosciences, Cardiff University, Cardiff CF10 3AX, UK; 2 Danau Girang Field Centre, Kota Kinabalu 88100, Malaysia; 3 Sabah Wildlife Department, Kota Kinabalu 88100, Malaysia and; 4 Sustainable Places Research Institute, Cardiff University, Cardiff CF10 3BA, UK

**Keywords:** Habitat fragmentation, hematology, health status, Malay civet, oil palm agriculture, serum biochemistry

## Abstract

Agricultural development is a major threat to global biodiversity, and effective conservation actions are crucial. Physiological repercussions of life alongside human-modified landscapes can undermine adaptable species’ health and population viability; however, baseline data are lacking for many wildlife species. We assessed the physiological status of a generalist carnivore, the Malay civet (*Viverra tangalunga*), persisting within an extensively human-modified system in Sabah, Malaysian Borneo. We characterized hematology and serum biochemistry panels from civets sampled across a mosaic landscape comprising tropical forest fragments and oil palm plantations. Intra-population variation in certain blood parameters were explained by expected biological drivers such as sex, age category and sampling season. Furthermore, we determined several erythrocyte measures, immune cell counts and dietary biochemistry markers significantly varied with proximity to oil palm plantation boundaries. These findings were supported by a case study, whereby blood profiles of GPS collared male civets were contrasted based on their exclusive use of forests or use of oil palm plantations. These data provide robust and valuable first insights into this species’ physiological status and suggest agricultural landscapes are impacting the persisting population.

## Teaser

Agriculture is a major threat to tropical biodiversity. We determined the first hematology and serum biochemistry panels for wild Malay civets persisting within a fragmented landscape. In addition to expected biological drivers of variation, select blood parameters predictably varied with proximity to oil palm agriculture.

## Introduction

Habitat loss, degradation and fragmentation pose significant threats to global biodiversity, and rates of species extinctions are at an all-time high (Ceballos *et al.,* 2017; IPBES, 2018). Species face suites of both global and local stressors from human activities, which combine to undermine the survival capabilities of many wildlife populations. Determining the explicit mechanisms of species loss following anthropogenic change is complicated, yet critical for the design and execution of effective conservation management strategies (Laurance *et al.,* 2012; Ripple *et al.,* 2017).

Conservation physiology aims to link individual physiological fitness to anthropogenically mediated population declines, often, and ideally, prior to the manifestation of measurable losses (Cooke *et al.,* 2013; Wikelski and Cooke, 2006). Some species display an apparently greater ecological resilience to anthropogenic pressures, at least when assessed by more traditional ecological metrics such as species presence or localized abundance counts (Miller *et al.,* 2015; Sih, 2013). Individuals, and by extension, populations, surviving within fragmented and degraded habitats can however be chronically stressed (Johnstone *et al.,* 2011), malnourished (Birnie-Gauvin *et al.,* 2017), immunosuppressed (Messina *et al.,* 2018), at elevated risk of pathogen or parasite exposure (Brearly *et al.,* 2013) or fail to successfully reproduce (Banks *et al.,* 2007). It is therefore critical to assess the physiological health of individuals, and by extension, species, persisting alongside the interface of natural and human-modified landscapes. Such assessments enable effective conservation actions to be crafted through the identification of otherwise cryptic threats to the long-term survival of a population (e.g. Cooke *et al.,* 2012; Madliger *et al.,* 2017).

The determination of wildlife health is a complex and developing field (Kindig and Stoddart, 2003; Stephen, 2014), and multiple metrics have evaluated species’ physiological responses to life in a changing world (Ellis *et al.,* 2012; Johnstone *et al.,* 2017; Madliger *et al.,* 2018). The evaluation of hematology and serum biochemistry profiles is one of the most informative assessments in determining the health and condition of wild species (Couch *et al.* 2017; Johnstone *et al.* 2017). Mammalian blood is a highly dynamic and reactive fluid composed of erythrocytes, leukocytes, thrombocytes and a suite of circulating pathophysiological markers such as minerals and hormones; all of these components can react to and reflect the overall status of an individual (Kerr, 2002). Natural variation in the absolute quantification of blood parameters collected from healthy individuals exists (Friedrichs *et al.,* 2012), and these reference ranges frequently depend upon intrinsic factors such as the sex, age and reproductive status of an individual (Keohane *et al.,* 2016). Clinically, changes in blood parameters away from relative baselines can be indicative of disease, malnutrition, toxicant exposure or chronic stress (Davis and Maney, 2018; Johnstone *et al.,* 2017; Maceda-Veiga *et al.,* 2015). Intrapopulation variation in blood parameters such as erythrocyte characteristics, white blood cell (WBC) counts, stress indices and lipid profiles of species persisting in fragmented and degraded habitats have quantified some of the physiological prices of persistence (e.g. García-Feria *et al.,* 2017; Irwin *et al.,* 2010; Johnstone *et al.,* 2012; May-Júnior *et al.,* 2009; Tyagi *et al.,* 2019). However, established blood profiles are still severely lacking for most wildlife species (Deem *et al.,* 2001), due at least in part to the invasive nature of sample collection and processing (Maceda-Veiga *et al.,* 2015). Thus, the collection and assessment of blood profiles of wild species are both warranted and crucial as the human impact continues to increase across the globe (Haddad *et al.,* 2015).

**Figure 1:**
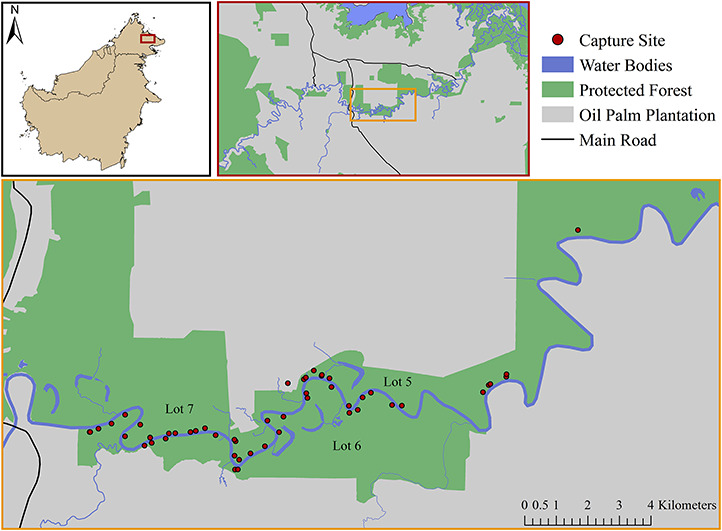
Malay civet (*Viverra tangalunga*) sampling sites from 2015–2019 across the Lower Kinabatangan Floodplain in eastern Sabah, Malaysian Borneo

The loss and degradation of Bornean lowland tropical rainforests via the establishment of oil palm (*Elaeis guineensis*) plantations pose a direct threat to the rich faunal diversity of the region (Meijaard *et al.,* 2018). Generalist species, such as long- and pig-tailed macaques (*Macaca fascicularis; M. nemestrina*), wild pigs (*Sus scrofa*), Asian water monitor lizards (*Varanus salvator*) and civets (Viverridae spp.) are observed within the agricultural matrix and in small patches of degraded forests alongside plantations; some researchers go so far as to postulate these species may thrive in such landscapes (Guerrero-Sanchez, 2019; Holzner *et al.,* 2019; Luskin *et al.,* 2017; Twining *et al.,* 2017). There are, however, scant, if any, studies specifically evaluating the physiological health status of these species within anthropogenically modified landscapes. Indeed, very little is known concerning the basic ecology of Bornean generalists, particularly the enigmatic small carnivores (Mathai *et al.,* 2016). One such species is the Malay civet (*Viverra tangalunga*), a 3–7 kg ground-dwelling, nocturnal and omnivorous small carnivore. This species is frequently documented within disturbed landscapes, including alongside agriculture (Jennings and Veron, 2009; Ross *et al.* 2016). There is evidence, however, that the species’ persistence within and adaptability to human-modified landscapes comes at an ecological cost; collared individuals using oil palm plantations hold significantly larger home ranges than those exclusively using remnant forest patches (Evans *et al.*, in review). There are currently no captive standardized reference intervals for this species within the Species360 database (Species360, 2019), or peer-reviewed reports of wild sampling efforts; thus, in order to more thoroughly determine the physiological repercussions of this species’ flexibility to human-modified ecosystems, baseline data are critically required.

Through the deployment of a multi-year trapping and sampling campaign, we evaluated the physiological status of Malay civets persisting within the agriculturally fragmented landscape of the Lower Kinabatangan Floodplain. We robustly describe, for the first time, the hematological and serum biochemistry parameters for a wild Bornean viverrid. This study further aimed to evaluate how variations in civet blood parameters were related to landscape and seasonal factors, while identifying and statistically controlling for natural physiological variation from the age and sex of an animal. Finally, we compared the results from the population-level modelling with the blood profiles of GPS collared adult male civets with known spatial movements.

## Methods

### Study site

This study was based in the Lower Kinabatangan Floodplain in eastern Sabah, Malaysian Borneo (Fig. 1). The climate of the area is considered humid tropical; daily temperatures ranged from 22–24°C during the study period. Mean (± SE) annual rainfall measured 2680 ± 210.5 mm, with wetter months typically spanning October through March. The mosaic landscape of the Floodplain comprises fragments of protected albeit degraded lowland forests surrounded by commercial oil palm plantations. Trapping occurred within the Lower Kinabatangan Wildlife Sanctuary, a series of 10 discontinuous lots totaling 270 km^2^ alongside the Kinabatangan River; animals were specifically sampled from lots 5–7. The protected area network includes an additional seven forest reserves, managed by the Sabah Forestry Department, and altogether covers a total area of 450 km^2^. These patches contain a mixture of dry lowland, semi-inundated, swamp and mangrove forests interspersed with grasslands (Abram *et al.,* 2014). The majority of the Floodplain has been converted into agricultural land use, particularly large-scale oil palm plantations (Abram *et al.,* 2014); as of 2014, over 98% of the remaining forested areas are within 1 km of an edge (Francis, 2017).

### Animal capture and sampling

Civet live trapping began in April 2015 and concluded in July 2019. Civets were captured using custom-built treadle-activated box traps (110 cm × 35 cm × 40 cm) and commercial spring-loaded traps (91 cm × 30 cm × 30 cm). Trap sets were carefully selected based on adequate canopy cover and high dry ground, and each was covered with foliage to provide additional shelter. We baited traps with audio lures and used cooking oil by 18:30 h. Sites were checked between 07:00 h and 08:00 h the following morning and were closed during the day to avoid diurnal captures of non-target species.

Upon capture, animals were carefully subdued within the trap with the aid of a purpose-built wooden squeeze door, whereupon a qualified veterinarian administered anesthesia via intramuscular injection. Animals were sedated with either 5 mg kg^−1^ tiletamine/zolazepam (Zoletil™, Virbac Laboratories, France) or a mixture of equal parts 1.5 mg kg^−1^ ketamine (Narketan™, Vétoquinol UK Limited, UK), xylazine (Ilium Xylazil™, Troy Laboratories PTY Limited, Australia) and tiletamine/zolazepam. In the case of the latter drug combination, the reversal agent yohimbine (Reverzine™, Bomac Pty Limited, Australia) was administered (1:1–1:3 xylazine volume: reversal volume ratio) once the animal was safely placed in a pet carrier to recover. All animal handling protocols and sampling procedures were approved by the Sabah Wildlife Department and the Sabah Biodiversity Centre (license ref. no: JKM/MBS.10000–2/2 JLD.6[8]). Our capture and GPS collar deployment techniques followed the guidelines set by the American Society of Mammalogists (Sikes *et al.,* 2016).

Throughout the sampling procedures, we monitored the animal’s vital signs (respiration rate, heart rate, rectal temperature and peripheral oxygen saturation [SpO_2_]) every 5 min. Once the anesthetic plane was deemed adequate, we collected ~1 ml kg^−1^ of blood from each individual via cephalic, brachial or femoral venipuncture (23-gauge/3-cc syringe or 25-gauge/1-cc syringe). Blood aliquots were immediately stored in plain and ethylenediaminetetraacetic acid (EDTA)-containing vacutainer tubes and placed in a cooler during field sampling. Whole blood was centrifuged ~4 h post-collection at 1000 rpm for 15 min (model EBA 21, Hettich Instruments, Tuttlingen, Germany). The serum was separated from the clot and frozen (−20°C), while the sample stored within EDTA was kept cool (~3–5°C) until sample transfer. We sent samples within 24–48 h from collection to Gribbles Pathology Laboratory Sdn Bhd (Sandakan, Sabah, Malaysia) for serum biochemistry and hematology analyses. This fully MS ISO 15189 accredited analytical laboratory is the leading local authority in handling biological samples and supplies reliable results to the hospitals and veterinarian clinics in the region. A total of 16 hematology and 23 serum biochemistry parameters were determined from civet blood samples (described in Supplementary Table 1).

During samplings, we conducted a complete physical examination of the animal, and weight, sex, age and reproductive status were evaluated. Age was scored by a combination of tooth condition, body size and signs of reproductive activity (females: nipple wear, lactation or palpably pregnant; males: testicular descent). The attending veterinarian determined pregnancy status by abdominal palpation. We assigned age categories as juvenile (small in size, presence of milk teeth), subadult (small in size, transitional dentition), adult (large body size, signs of active reproduction, mature dentition) or old (slight body condition despite large size, poor or worn teeth); all assessments were made by the same observer (M. Evans). A radio-frequency identification microchip was subcutaneously implanted between the shoulder blades of each anesthetized individual (Trovan Ltd, UK). We fitted GPS collars on select adult males to determine individual patterns of habitat use. Briefly, these units collected hourly nocturnal locations from tagged animals for an average of 120 nights. For tag specifications and performance, see details provided in Evans *et al.* (2016); spatial data handling protocols and home range results were reported in Evans *et al.* (in review).

### Data analysis

We assessed the effects of Malay civet sex, age category, sampling season and oil palm plantation proximity on recorded blood parameters with generalized linear models in R (version 3.5.0, R Core Team, 2018). Individuals were binned into a two-level classification scheme of immature (juvenile + subadult) or mature (adult + old), and sampling season was delineated as wet (October–March) or dry (April–September) months. The distance from the capture site of an animal to the nearest oil palm plantation was determined with ArcGIS 10.1 (ESRI, 2011) from digitized Google Earth Pro satellite imagery; these measurements only determined accessible agriculture, as defined by those plantations on the same riverbank as civet capture site. This statistical approach makes no assumptions regarding whether the magnitude of each parameter is considered healthy or normal for this species; this process instead robustly evaluated the drivers behind the observed parameter variations within the sampled population. All individuals were visibly healthy (in good body condition with no clinically apparent symptoms of illness); for individuals with multiple capture events, only blood parameters from the first sampling event were included in model development.

The specific blood parameter data type determined each model family, and link functions were selected based on AIC minimization and best normalization of model residual plots Supplementary Table 1). Count data were first modeled with Poisson model families; however, as all were found to be over-dispersed (over-dispersion statistic >20; Thomas *et al.,* 2017), models were instead fit with negative binomial structures with the *MASS* package (Venables and Ripley, 2002). Proportional datasets were modeled using binomial generalized linear models fitted with ‘logit’ link functions (Thomas *et al*., 2017). Lastly, due to reported values below laboratory detection limits for uric acid, bilirubin and GGT, these data were conservatively binned into binomial classes; models thus evaluated the factors influencing values below versus above the limit of detection within the sample population.

The most parsimonious model for each blood parameter was determined by multi-model inference, whereby candidate model structures were generated and small-sample size corrected Akaike’s Information Criteria (AICc) values compared using the ‘dredge’ function in the *MuMIN* package (Bartón, 2018). The top models were selected as those with a ∆AICc <2 and were averaged using the natural average method to more wholly account for uncertainty in the model structure selection process (Burnham and Anderson, 2002). Prior to model averaging, oil palm proximity measurements were standardized to a mean of 0 and standard deviation of 0.5 to allow for direct comparison between terms (Grueber *et al*., 2011).

GPS collared male Malay civets were grouped according to the habitat types accessed throughout their collaring periods (‘forest only’ or ‘mixed’ habitat individuals, per Evans *et al*., in review). Mixed habitat individuals accessed both protected forests and industrial oil palm plantations in their total home ranges. Blood parameters of these groups were compared using either t-tests or Mann–Whitney U tests, as appropriate based on normality of each parameter.

## Results

### Total population

We captured and collected samples from 58 Malay civet individuals within the Lower Kinabatangan Floodplain (Fig. 1). Hematology profiles were successfully established for 51 unique Malay civets (25 males, 26 females). Serum biochemistry panels were successfully determined for 56 unique Malay civets (25 males, 31 females). Of these, 17 samples were removed from potassium, calcium and alkaline phosphatase datasets; these values were statistically extreme in a pattern consistent with suspected K-EDTA contamination during sample collection (Bowen and Remaley, 2014).

A total of seven hematology and eight biochemistry parameters were significantly influenced by sex, age category, sampling season or proximity to oil palm plantation; the remaining parameters were not significantly related to any of these variables (Tables 1–2). Summary descriptions of the blood parameters of the sampled population, partitioned and annotated as appropriate given the GLM results, are displayed in Supplementary Tables 2–4.

Only hematology parameters significantly varied between civet sexes (Tables 1–2). Hemoglobin (Haem) concentration was significantly elevated in males compared to females. Total red blood cell (RBC) counts and packed cell volume (PCV) values were also elevated in males compared to females, and in animals trapped in the dry compared to the wet season. In contrast, females expressed significantly elevated lymphocyte (Lymp) counts compared to males, as did immature compared to mature individuals.

Civet age was the only biological term that significantly described variations in serum biochemistry parameters (Tables 1–2). Phosphate (P) and alkaline phosphatase (AlkPho) levels were significantly elevated in immature compared to mature civets. Mature animals, in contrast, expressed significantly higher creatinine (Creat), total protein (TotProt) and globulin (Glo) levels than immature animals.

**Table 1:** Standardized parameter estimates for statistically significant averaged models describing variation in Malay civet (*Viverra tangalunga*) hematology and serum biochemistry parameters. NS denotes statistically non-significant (*P* > 0.05) terms. Bold terms emphasize significant variables. SeasonW = wet season; SexM = male; Plant = distance to nearest oil palm plantation; AgecatM = mature civets. Intercept is the standardized reference level for factorized predictor variables (e.g. dry season, female, immature). Shorthand notation for response variables matches those in Supplementary Table 1. ^#^denotes a non-averaged final model (i.e. there were 0 additional models whereby ∆AICc <2)

**Parameter**	**Variable**	**Estimate**	**Std. Error**	**z value**	***P* value**
Haem	(Intercept)	110.52	2.26	47.68	<0.00001
	SeasonW	−4.906	5.28	0.919	NS
	**SexM**	18.104	4.531	3.896	<0.001
RBC	(Intercept)	797.49	35.93	21.70	<0.00001
	**SeasonW**	−78.73	35.19	2.18	<0.05
	**SexM**	105.36	34.74	2.960	<0.005
	AgecatM	−9.34	27.33	0.34	NS
PCV^#^	(Intercept)	−0.362	0.046	−7.909	<0.00001
	**SexM**	0.261	0.057	4.591	<0.0001
	**SeasonW**	−0.153	0.058	−2.622	<0.01
MCHC	(Intercept)	5.556	0.00848	639.35	<0.00001
	**Plant**	0.05032	0.01712	2.866	<0.005
	SeasonW	0.00708	0.01402	0.497	NS
WBC	(Intercept)	7.084	0.078	88.84	<0.00001
	**Plant**	0.1651	0.0744	2.164	<0.05
	SeasonW	−0.0458	0.0736	0.614	NS
	AgecatM	−0.0343	0.0727	0.465	NS
Neut	(Intercept)	6.702	0.076	86.27	<0.00001
	**Plant**	0.267	0.1035	2.513	<0.005
	SeasonW	−0.138	0.0953	0.592	NS
	SexM	0.0412	0.0839	0.484	NS
Lymp	(Intercept)	5.916	0.167	34.59	<0.00001
	**AgecatM**	−0.3622	0.164	2.145	<0.05
	**SexM**	−0.3335	0.135	2.398	<0.02
	SeasonW	−0.0417	0.100	0.409	NS
HDL	(Intercept)	0.7659	0.02779	26.931	<0.0001
	**Plant**	−0.1500	0.05839	2.513	<0.02
	AgecatM	0.05666	0.07082	0.791	NS
	SexM	−0.07444	0.06924	1.063	NS
Ratio	(Intercept)	0.4615	0.01668	27.048	<0.0001
	SeasonW	0.02843	0.03753	0.75	NS
	**Plant**	0.10402	0.03403	2.987	<0.005
	SexM	0.00933	0.02352	0.391	NS
Ur	(Intercept)	8.2232	0.2813	28.55	<0.0001
	**Plant**	2.0806	0.6012	3.382	<0.001
	SexM	0.5154	0.6237	0.817	NS
	SeasonW	−0.2304	0.4476	0.508	NS
Creat	(Intercept)	4.1823	0.0269	151.72	<0.0001
	**AgecatM**	0.1559	0.0647	2.354	<0.02
	SeasonW	−0.0169	0.0407	0.408	NS
P	(Intercept)	0.4408	0.03258	13.22	<0.0001
	**AgecatM**	−0.3074	0.08171	3.681	<0.0005
	SeasonW	−0.0865	0.08291	1.02	NS
	Plant	−0.0572	0.07029	0.804	NS
TotProt	(Intercept)	79.537	0.9338	83.23	<0.0001
	**AgecatM**	6.5099	2.1661	2.938	<0.005
	Plant	1.9261	2.1472	0.887	NS
	SexM	−0.3715	1.1369	0.323	NS
	SeasonW	0.5751	1.4097	0.401	NS
Glo	(Intercept)	50.73	0.9665	51.208	<0.0001
	**AgecatM**	6.671	2.1227	3.068	<0.005
	SexM	−3.041	2.3626	1.270	NS
	Plant	2.026	2.3921	0.839	NS
AlkPho^#^	(Intercept)	4.0505	0.2600	15.581	<0.0001
	**AgecatM**	−0.7534	0.2907	−2.592	<0.02

**Table 2:** Top candidate (∆AICc <2) model structures included in model averaging, log-likelihood (Log*L*), Akaike’s Information Criterion with the small sample bias adjustment (AICc) and Akaike weights (*wi*) for predicting relationships between standardized independent biological and spatial variables and Malay civet hematology and serum biochemistry parameters. Season = dry or wet capture season; Sex = male or female civet; Agecat = immature or mature civet; Plant = distance from capture site to nearest accessible oil palm plantation. Refer to Supplementary Table 1 for parameter shorthand reference. ^#^denotes a non-averaged final model (i.e. there were 0 additional models whereby (∆AICc <2))

**Parameter**	**Candidate model**	***df***	**Log*L***	**AICc**	**ΔAICc**	***wi***
Haem	Season + Sex	4	−213.14	435.14	0	0.6
	Sex	3	−214.72	435.96	0.81	0.4
RBC	Season + Sex	4	−317.7	644.28	0	0.72
	Agecat + Season + Sex	5	−317.41	646.15	1.87	0.28
PCV^#^	Season + Sex	3	−163.11	326.46	0	1
MCHC	Plant	3	−211.25	429.02	0	0.63
	Plant + Season	4	−210.60	430.08	1.06	0.37
WBC	Plant	3	−363.93	734.36	0	0.39
	Plant + Season	4	−363.15	735.17	0.81	0.26
	Agecat + Plant	4	−363.53	735.93	1.57	0.18
	Agecat + Plant + Season	5	−362.35	736.04	1.68	0.17
Neut	Plant	3	−346.56	699.65	0	0.38
	Plant + Season	4	−345.70	700.31	0.67	0.27
	Plant + Sex	4	−345.95	700.82	1.17	0.21
	Plant + Season + Sex	5	−345.08	701.56	1.91	0.14
Lymp	Agecat + Sex	4	−296.5	601.91	0	0.69
	Agecat + Season + Sex	5	−296.06	603.51	1.59	0.31
HDL	Agecat + Sex + Plant	5	−29.61	70.43	0	0.38
	Sex + Plant	4	−31.05	70.88	0.45	0.3
	Agecat + Plant	4	−31.66	72.11	1.68	0.16
	Plant	3	−32.87	72.21	1.78	0.16
Ratio	Season + Plant	4	12.35	−15.91	0	0.33
	Plant	3	10.94	−15.42	0.49	0.26
	Season + Sex + Plant	5	12.83	−14.47	1.45	0.16
	Sex + Plant	4	11.37	−13.96	1.95	0.12
	Agecat + Plant	4	11.36	−13.93	1.98	0.12
Ur	Sex + Plant	4	−113.63	236.07	0	0.36
	Plant	3	−115.12	236.70	0.64	0.26
	Season + Sex + Plant	5	−113.02	237.26	1.19	0.20
	Season + Plant	4	−114.39	237.58	1.51	0.17
Creat	Agecat	3	−219.3	445.07	0	0.68
	Agecat + Season	4	−218.9	446.59	1.52	0.32
P	Agecat + Season + Plant	5	−19.88	50.95	0	0.37
	Agecat + Season	4	−21.29	51.36	0.41	0.30
	Agecat + Plant	4	−21.76	52.31	1.35	0.19
	Agecat	3	−23.14	52.75	1.79	0.15
TotProt	Agecat + Plant	4	−186.91	382.60	0	0.29
	Agecat	3	−188.21	382.89	0.29	0.25
	Agecat + Sex + Plant	5	−186.24	383.69	1.09	0.17
	Agecat + Season + Plant	5	−186.41	384.01	1.41	0.14
	Agecat + Season	4	−187.67	384.12	1.52	0.14
Glo	Agecat + Sex + Plant	5	−174.08	359.43	0	0.53
	Agecat + Sex	4	−176.00	360.83	1.40	0.26
	Agecat	3	−177.41	361.31	1.88	0.21
AlkPho^#^	Agecat	3	−148.79	297.70	0	1

Finally, the distance between oil palm plantation boundaries and capture sites significantly related to variations in both hematology and biochemistry parameters (Tables 1–2). Malay civets captured closer to plantations expressed significantly depressed mean corpuscular hemoglobin concentration (MCHC), WBC counts and neutrophil (Neut) counts. Measured high density lipoprotein (HDL) decreased as the distance between the capture site and plantation increased. In contrast, civet total cholesterol to HDL ratio (Ratio) and urea (Ur) levels increased with increasing distance from agriculture.

### GPS collared males

**Figure 2:**
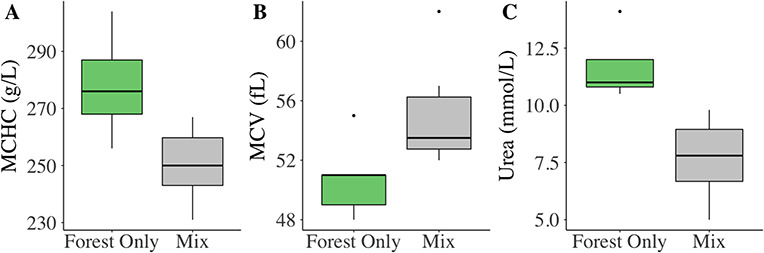
Blood parameters of GPS collared adult male Malay civets (*Viverra tangalunga*) using only forests (n = 5) or forest and oil palm plantations (mix, n = 8) that showed significant differences in (A) mean corpuscular hemoglobin concentration (MCHC); (B) mean corpuscular volume (MCV); and (C) urea concentrations based on habitat utilization. (Mann–Whitney U/t-test at *P* < 0.05)

In total, we successfully collected blood from 13 GPS collared adult males. Malay civets that did not access oil palm (n = 5) expressed significantly elevated MCHC, decreased mean corpuscular volume and elevated serum urea concentrations compared to those that used both forests and oil palm plantations (n = 8; Fig. 2; Supplementary Tables 5–6).

Malay civets that used only forests expressed significantly elevated MCHC compared to those individuals that entered agriculture (t = 3.213, df = 11, *P* < 0.01). Specifically, forest only males expressed a mean MCHC value 27 g/L greater than those that entered oil palm plantations. Civet MCV significantly varied between civet habitat usages (Mean ± SD; forest only animals: 50.8 ± 2.7 fL; mix habitat animals: 54.9 ± 3.4 fL; t = −2.264, df = 11, *P* < 0.05). Civets that resided solely in the forests expressed significantly elevated urea concentrations compared to those individuals that entered the agriculture (t = 4.393, df = 11, *P* < 0.01); forest only animals expressed a mean urea concentration 4.01 mmol/L greater than those that entered oil palm plantations.

## Discussion

This study provides insights into the physiological status of a small carnivore species persisting within the agriculturally fragmented Lower Kinabatangan Floodplain. By conducting the longest running field study of this species to date, a multi-facetted narrative regarding the risks and possible rewards of persistence in the mosaic Kinabatangan landscape is presented. We report the first hematology and serum biochemistry profiles for this species; the establishment of these values, although not to be directly interpreted as healthy reference intervals in the clinical sense, provides a foundation for future studies to contextualize their findings. Civet blood parameter measurements were influenced by sex, age, sampling season and proximity to oil palm plantation; these findings were supported by a case study approach evaluating the blood profiles of GPS collared civets with known spatial behaviours. Modeling results suggest intrapopulation variation in blood parameters is attributed, in addition to expected physiological drivers, to anthropogenically mediated processes.

### Biological effects

At the population level, civet blood parameter measurements were influenced by biological factors, reinforcing the importance of acknowledging these traits in wild animal health assessments. Males expressed elevated hemoglobin levels, RBC counts and PCV compared to females. These parameters are frequently naturally elevated in male mammals (Iberian lynx, *Lynx pardinus*, Beltrán *et al.,* 1991; grizzly bear, *Ursus arctos*, Brannon, 1985; Tasmanian devil, *Sarcophilus harrisii*, Hope and Peck, 2016; reviews by Kerr, 2002; Murphy, 2014); this is largely attested to the stimulating effects of sex hormones, specifically androgens, on RBC production processes (Kerr, 2002; Zitzmann and Nieschlag, 2004). RBC count and PCV were further influenced by capture season, whereby individuals sampled in the dry season expressed elevated values relative to those captured in the wetter season. This likely suggests civet hydration status decreases during the dry season, as both PCV and RBC measurements are clinically elevated through a reduction in circulating plasma volume in dehydrated individuals (Kerr, 2002; Nayak *et al.,* 2012). Interestingly, these were the only parameters for the species to vary with season, in contrast to other carnivore blood profile studies (e.g. Eastern quoll, *Dasyurus viverrinus*, Fancourt and Nicol, 2019; Tasmanian devil, Stannard *et al*., 2016; brown hyena, *Parahyaena brunnea*, Wiesel *et al.,* 2018). This could be due to the lack of environmentally driven breeding season for the species (indeed, we failed to detect any discernable temporal patterns in female pregnancy rates during our sampling efforts [Evans, 2019]). Alternatively, our simplified binary categorization of the rainfall patterns in lowland rainforests may not fully encompass the nuances in daily weather patterns relevant to blood metrics.

With respect to immune system parameters, female Malay civet lymphocyte counts were elevated compared to males, as were levels for immature civets. There is established clinical evidence that mammalian immunology naturally varies between males and females (Klein and Flanagan, 2016). Mechanistically, testosterone levels of male civets may suppress lymphocyte production rates relative to those of female civets (Foo *et al.,* 2017). Alternatively, circulating estrogen and progesterone could biochemically enhance female civets’ adaptive immune responses (Foo *et al.,* 2017; Klein, 2000). Lower male lymphocyte counts have been similarly recorded in Tasmanian devils (Hope and Peck, 2016). Further, elevated lymphocyte counts are reported in young and developing animals (Kerr, 2002; Nussey *et al.*, 2011), suggesting civet immune systems may follow patterns expected from targeted studies. Alternatively, these results may be related to reproductive activity; indeed, as early term pregnancy was unable to be determined for sampled civets, there could be a cryptic exacerbating effect of pregnancy on adult female lymphocyte counts (Roved *et al.,* 2017).

Similarly, the elevation of phosphate and alkaline phosphatase concentrations in immature Malay civets compared to adults was not unexpected and can be explained by physiological growth processes. Both the mineral and enzyme increase in circulation during bone growth and development processes occurring in young individuals (Fernandez and Kidney, 2007). This age effect has been documented in many wild mammalian carnivores (common palm civet, *Paradoxurus hermaphroditus*, Ahmad *et al.,* 2017; grizzly bear, Brannon, 1985; Florida panther, *Puma concolor coryi*, Dunbar *et al*., 1997; Iberian lynx, García *et al.,* 2010; Tasmanian devil, Hope and Peck, 2016; Peck *et al*., 2015; Culpeo fox, *Lycalopex culpaeus*, Rubio *et al*., 2014; grey wolf, *Canis lupus*, Seal *et al*., 1975; brown hyena, Wiesel *et al*., 2018). Further, lower globulin and total protein measurements in immature relative to mature individuals are likely due to the development of immune responses and hepatic systems of maturing animals (Kerr, 2002). This is similarly in agreement with records in other carnivores (Amur leopard, *Panthera pardus orientalis*, Bodgener and Lewis, 2017; grizzly bear, Brannon, 1985; red panda, *Ailurus fulgens*, Burrel *et al.,* 2018; Tasmanian devil, Hope and Peck, 2016; Peck *et al.*, 2015; Stannard *et al.,* 2016; Indian leopard, *P.p. fusca*, Shanmugam *et al.*, 2017; and brown hyena, Wiesel *et al.,* 2018). The documented elevation in mature civet creatinine levels likely relates to differences in muscle mass between adult and growing individuals (Kerr, 2002), as creatinine is a product of muscle catabolism (Delanaye *et al.,* 2017). We suggest it is unlikely there is evidence of age-driven variation in Malay civet renal performance, as urea levels did not vary with animal age, as would be clinically expected from renal complications. Similar patterns were documented in Iberian lynx (Beltrán *et al.,* 1991; García *et al*., 2010), Amur leopard (Bodgener and Lewis, 2017), polar bear (*Ursus maritimus,*  Fry *et al.,* 2019) and Chinese pangolin (*Manis pentadactyla,*  Khatri-Chhetri *et al.,* 2015).

### Landscape effects

In addition to the biological variations in Malay civet blood parameters, we documented landscape-level effects on carnivore physiology. MCHC significantly varied in relation to landscape configuration; civets captured closer to oil palm plantations expressed significantly lower MCHC compared to those sampled farther from agriculture. As the MCHC value directly relates to the oxygen transport potential of blood, a relative decrease of this index can clinically indicate hypochromic anemia (Johnstone *et al.,* 2017). A decrease in MCHC can relate to difficulties in hemoglobin synthesis, which is most often attributed to insufficient iron levels (Keohane *et al.,* 2016); indeed, iron deficiencies in wild carnivores persisting in degraded habitats have been reported (grey wolf, DelGiudice *et al.,* 1991; howler monkey, *Alouatta pigra*, García-Feria *et al.,* 2017; maned wolf, *Chrysocyon brachyurus*, May-Júnior *et al.,* 2009). Clinically, anemia resulting from an iron deficiency generates both hypochromic and microcytic (reduced MCV) RBC profiles; the population-level Malay civet MCV values, however, did not vary with proximity of oil palm plantation. Additional research into the specific iron status of wild civets within the fragmented landscape is thus warranted, such as specific serum iron, transferrin levels and ferritin concentrations (Hoffbrand *et al.,* 2006). Alternatively, exposure to certain toxins can result in anemia related to hemoglobin concentrations (Katavolos *et al.,* 2007; Serieys *et al.,* 2018); it is possible Malay civets nearer to oil palm are being exposed to such a pollution source.

Malay civet leukocyte profiles were significantly related to proximity to oil palm plantations, as individuals captured closer to agriculture expressed lower circulating neutrophil and total WBC counts than those sampled farther away. Given the lack of species-specific reference intervals for normal or baseline leukocyte counts, this variation can be interpreted in two ways (Salvante, 2006): (1) those individuals farther from oil palm plantations were actively responding to elevated infections, and thus the lower leukocyte values in civets sampled near oil palm represent normal immune system baselines; or (2) those individuals sampled farther from oil palm plantations were of optimal immune condition, and thus the lower leukocyte values near plantations were indicative of immune impairment. Relative leukopenia and neutropenia have been documented in other species exposed to various human disturbances; these studies all concluded anthropogenic activities depress the immunological status of persisting wildlife (bat spp., Allen *et al.*, 2009; Seltmann *et al.,* 2017; diademed sifaka, *Propithecus diadema*, Irwin *et al.,* 2010; agile antechinus, *Antechinus agilis*, Johnstone *et al.,* 2012; bobcat, *Lynx rufus*, Serieys *et al.,* 2018; reviewed by Messina *et al.,* 2018). The suggested biochemical mechanisms for such reactive immunosuppression remain largely hypothetical; the physiological upkeep of a functional innate cellular immune system is metabolically costly, so decreases in circulating neutrophils may be a (mal)adaptive response to other energetic demands associated with persistence in degraded landscapes (Pfeifer *et al.,* 2017). Exposure to immunosuppressing chemicals, such as pesticides or heavy metals, is also often elevated in disturbed habitats (Selgrade, 2007; Serieys *et al.*, 2018). The results from this study suggests Malay civets captured nearer to agriculture express lowered innate immune functionality than those in less agriculturally disturbed areas, which may carry significant implications for these individuals’ susceptibility to pathogens and reproductive capabilities (Acevedo-Whitehouse and Duffus, 2009; French *et al.,* 2007).

Civet lipid profiles varied with proximity to oil palm plantations, suggesting diet may vary across the landscape. Animal lipid profiles respond to food consumption, be it via changes in the selection of specific food items or changes in the nutritional quality of food, particularly fat and carbohydrate content (Shanmugam *et al.,* 2011; Wiesel *et al.,* 2018). Surprisingly, the ratio of total cholesterol to HDL–cholesterol levels were lower, and HDL–cholesterol higher, in civets captured nearer to oil palm plantations; such a lipoprotein balance is clinically indicative of a reduced risk of cardiovascular disease in mammals (Bruss, 2008). Interestingly, nutritional research claims consumption of oil palm products increases HDL–cholesterol in both humans and laboratory rats (Dauqan *et al.*, 2011; Sun *et al.*, 2015). Indeed, this is often a significant selling point of marketing campaigns lauding oil palm as a ‘healthier’ vegetable oil product (Kushairi *et al.,* 2018). Guerrero-Sanchez (2019) recorded similar patterns in Asian water monitor lizards captured within the Kinabatangan plantation landscape. Malay civets are flexible dietary generalists and have been observed ingesting both oil palm fruits and small mammals within plantations (Joscelyne; unpublished data; Guharajan *et al.,* 2019). Alternatively, there is evidence that circulating HDL–cholesterol can be increased by elevated cardiovascular exercise (Kraus *et al.,* 2002). As GPS collared male civets accessing oil palm agriculture held larger home ranges than those individuals that remained within the forest (Evans *et al.,* in review), the lipid profiles in this sampled population may reflect the more demanding foraging activities by animals persisting alongside agriculture.

Malay civet dietary flexibility across the Lower Kinabatangan Floodplain is further indicated by the positive relationship between blood urea concentration and distance from capture site to oil palm plantation. Serum urea is traditionally measured as part of a renal function panel, whereby elevation indicates dysfunction (Kerr, 2002). Civet creatinine levels, however, did not predictably vary with plantation proximity. Creatinine is the clinically more specific marker used in the diagnosis of renal dysfunction (Braun and Lefevre, 2008), thus this spatial variation in civet urea is most likely due to a factor not directly related to renal function. An increase in protein consumption can elevate circulating urea levels, as urea is a waste product of amino acid breakdown (Kerr, 2002). This suggests Malay civets captured closer to oil palm plantations may have diets lower in protein relative to those captured deeper in the forest. Depressed blood urea values measured from wildlife in fragmented and degraded landscapes have been attributed to deficiencies in dietary protein intake (maned wolf, Curi *et al.,* 2015; howler monkey, García-Feria *et al.,* 2017; diademed sifaka, Irwin *et al.,* 2010; bobcat, Serieys *et al.,* 2018). Further targeted research into Malay civet dietary habits relative to landscape composition is strongly required to untangle these findings; genetic assessments of civet feces (e.g. Cancio *et al.,* 2017) could evaluate the degree of omnivory in the Kinabatangan civets relative to landscape composition.

### Case study: GPS collared civets

The differences in blood parameters of the GPS collared Malay civets compared well to the modeled landscape patterns reported from the sampled population. The similarly depressed MCHC in males utilizing oil palm plantations compared to those remaining exclusively in the forest adds further weight to the above hypothesis of landscape-facilitated anemia in civets associating with agriculture*.* Intriguingly, this case study reported not just relative hypochromia within the animals accessing agriculture, but also a relative macrocytic MCV profile (Keohane *et al.,* 2016); when considered together, these results may provide evidence of regenerative anemia within the population (Johnstone *et al.,* 2017). This type of anemia is frequently the result of chronic stressors experienced by wild populations; following blood loss (e.g. parasitism, injury), exposure to certain toxins or elevation of circulating stress hormones, there is an increased release of reticulocytes (immature erythrocytes) from the bone marrow to keep up with the body’s demand for RBCs (Johnstone *et al.,* 2011). Reticulocytes are, however, both larger in size and less capable of producing hemoglobin than fully mature erythrocytes, which could explain the relative macrocytic and hypochromic characteristic patterns described in these GPS collared animals (Doig, 2016; Fisher and Crook, 1962; Keohane *et al.,* 2016). An examination of civet reticulocyte counts would provide more conclusive evidence of spatially-mediated regenerative anemia. Similar findings of anemia related to stress or parasite infections have been documented in other wild species (agile antechinus, Johnstone *et al.,* 2012; hedgehog, *Erinaceus europaeus*, Pfäffle *et al.,* 2009; Brazilian carnivore spp., Santos *et al.,* 2018); further research is warranted, particularly evaluating if the scale of measured differences is indicative of physiological detriment to a given individual, such as diminished reproductive success or dispersal survival (e.g. Vitousek *et al.,* 2014; Wingfield and Sapolsky, 2003).

In contrast, the leukocyte profiles of GPS collared civets did not reflect the population-level patterns of relative neutropenia-mediated leukocyte variation in individuals sampled nearer to oil palm plantations; however, collared individuals that accessed them expressed lower circulating monocyte counts relative to forest only civets, although this difference was not statistically significant. This does not immediately negate the hypothesis that oil palm agriculture may facilitate population-wide variations in immune function; these findings may indicate a shift in the mode of the condition from neutrophils to monocytes in this specific case. Confounding effects of stress, toxins and acute infections can all impact immune functioning (Davis *et al.,* 2008; Repetto and Baliga, 1997). Given the relatively small sample size of collared animals paired with the complexity of intraspecific immune system responses (Roquiz *et al.,* 2016), we recommend further research into the specific biochemical processes that may result in landscape-mediated patterns of immunosuppression.

Lastly, urea values were significantly decreased in civets utilizing oil palm plantations compared to those that remained exclusively in the forest. This is similarly consistent with the population-level model findings, suggesting there may be dietary or lipid metabolism differences between the spatial groups. The collared males did not demonstrate any differences in measured HDL concentration or total cholesterol to HDL ratio; this may be similarly due to the small sample size.

As previously stated, there are no available reference intervals from Malay civets or closely related phylogenetic proxies against which to contextualize the magnitude of our results. The implications of this are 2-fold: it is possible our discussed concerns are not clinically indicative of individual fitness repercussions; or it is possible the population as a whole expresses abnormal blood profiles, and thus we under report the extent of the dangers facing this species. For example, the lack of spatial trends in liver enzymes could be due to the fact that the Kinabatangan population displays elevated enzyme activity indicative of liver damage, regardless of distance to agriculture. These findings further highlight the importance, and difficulty, of determining species-specific reference intervals. Widening the extent of sampling efforts to include captive animals and populations in less anthropogenically disturbed landscapes would begin to more fully contextualize the physiological threats oil palm agriculture may pose to this flexible species.

Overall, several logistical considerations were unable to be controlled and may influence the reported blood profiles for the species. First, it is important to acknowledge these animals were captured in traps at a remote field site. This process intensifies the acute stress experienced by an individual, and we were unable to control for the possible effects of varied capture, animal and sample handling times on blood parameter measurements. There is evidence that wild animal blood profiles can be influenced by trapping and anesthetic methodologies (Brockman, 1981; Santos *et al.,* 2015), and although the parameters assessed here are less temporally sensitive than others such as hormone biomarkers, future comparative research should bear in mind the methodology employed in our study. Although all the animals included in this study were visually healthy, additional factors such as fasting status, endoparasite load, pathogen infection and pregnancy were not assessed, and thus unable to be accounted for, in this study design. Lastly, although utmost care was taken to minimize sample degradation due to improper storage temperatures or delays in clinical assessment, this study was conducted at a remote location without access to stable cold storage options such as liquid nitrogen, so some samples may cryptically be of subpar quality.

Despite these considerations, this study has provided evidence of landscape-facilitated variation in RBC, immune and biochemical dietary indicators within the Malay civet population of the Lower Kinabatangan Floodplain. This first evaluation of civet hematology and biochemistry profiles has provided a powerful reference toolkit for the rapid assessment of the physiological status of this adaptable species in a fragmented ecosystem. Most notably, we highlight the potential physiological costs of carnivore persistence alongside oil palm agriculture, which may undermine the long-term population viability of this species.

## Supplementary Material

Supplementary_Tables_Rev_coaa127
