# Supplementary material for: Physiological implications of life at the forest interface of oil palm agriculture: blood profiles of wild Malay civets (Viverra tangalunga)
Source: Conserv Physiol. 2020 Dec 30;8(1):coaa127. doi: 10.1093/conphys/coaa127 (PMC7772617; doi:10.1093/conphys/coaa127)
Supplement: Supplementary_Tables_Rev_coaa127 [file supplementary_tables_rev_coaa127.doc]

**Supplementary Materials**

**Supplementary Table 1**: Haematology (red) and serum biochemistry (blue) parameters determined for wild Malay civets (*Viverra tangalunga*) sampled in the Lower Kinabatangan Floodplain, 2013–2019.

| **Group** | **Parameter, Shorthand** | **Unit** | **Description** | **Source** | **Model Family, Link^*^** |
| --- | --- | --- | --- | --- | --- |
| **Erythrocyte (Red Blood Cell)**  **Parameters** | Haemoglobin,  Haem | g/L | Globular protein within erythrocytes used to transport O_2_ from lungs to tissues & CO_2_ from tissues to lungs; indicator of physiological status and anaemias | Keohane, 2016 | Gamma, Identity |
|  | Red blood cell count, RBC | /L | Total count of circulating erythrocytes within a volume of whole blood | Clark and Hippel, 2016 | Negative Binomial, Identity |
|  | Packed cell volume, PCV | L/L | Volume of packed red blood cells within a volume of whole blood. Also called hematocrit; can indicate anaemia | Clark and Hippel, 2016 | Binomial, Logit |
|  | Mean corpuscular volume, MCV | fL | Erythrocyte index equates to average volume of an erythrocyte. Aids in classification of anaemias | Clark and Hippel, 2016 | Gamma, Log |
|  | Mean corpuscular hemoglobin, MCH | pg | Erythrocyte index equates to average weight of haemoglobin per erythrocyte | Clark and Hippel, 2016 | Gamma, Log |
|  | Mean corpuscular hemoglobin concentration, MCHC | g/L | Erythrocyte index equates to average concentration of hemoglobin per erythrocyte. Aids in diagnosis of anaemias | Clark and Hippel, 2016 | Gamma, Log |
|  | Red cell distribution width, RDW | % | Quantitative assessment of the degree of variation in size of circulating erythrocytes. Assists in classification of anaemias and changes in erythrocyte morphology | Longanbach and Miers, 2016 | Binomial, Logit |
| **Leukocyte (White Blood Cells) Parameters** | Total, WBC | /L | Total circulating white blood cells (leukocytes) in 1 L of blood; general assessment of immune functioning | Roquiz *et al*., 2016 | Negative Binomial, Log |
|  | Neutrophil count,  Neut | /L | Granular leukocyte acts as primary phagocytic cell responding to infection, inflammation, and stress. Generally most abundant in circulation | Davis *et al.*, 2008 | Negative Binomial, Log |
|  | Lymphocyte count, Lymp | /L | Agranular leukocyte involved in various immune functions, including modulation of immune system and immunoglobulin production | Davis *et al.*, 2008 | Negative Binomial, Log |
|  | Monocyte count,  Mono | /L | Agranular & long-lived phagocyte associated with defense against infections and bacteria. Usually low circulating numbers | Roquiz *et al.*, 2016 | Negative Binomial, Sqrt |
|  | Eosinophil count,  Eos | /L | Granular leukocyte responds to inflammation and defense against helminth parasites. Usually low circulating numbers | Roquiz *et al.*, 2016 | NA |
|  | Basophil count,  Baso | /L | Granular leukocyte; functionality less understood; involved in allergies and inflammation. Usually low circulating numbers or absent | Roquiz *et al.*, 2016 | NA |
|  | Neutrophil:Lymphocyte ratio, N:L | - | Marker of stress responses in mammalian species, scales directly with stressor magnitude and glucocorticoid levels | Davis *et al.*, 2008 | Gamma, Sqrt |
| **Clotting** | Platelet count,  Plat | /L | Nonnucleated circulating blood cell that responds to blood vessel damage and commences clotting process | Fritsma, 2016 | Negative Binomial, Identity |
| **Glucose** | Glucose,  Gluc | mmol/L | Sugar monomer in blood from ingested carbohydrates; strongly influenced by fasting status, diseases like diabetes, and some relation to stress | McCowen *et al.*, 2001 | Gamma, Identity |
| **Lipids** | Total cholesterol,  Tchol | mmol/L | Lipid essential for normal function of cells, including cell membrane formation and hormone development. Transported within body by HDL and LDL. Excess can cause vascular disease | Ishigame *et al.*, 2006 | Gamma, Log |
|  | Triglyceride,  Tri | mmol/L | Lipid circulating in blood and main component of fat tissue. Transported by very low-density lipoproteins. High levels associated with cardiovascular disease and dietary intake | Kalinski *et al.*, 2017 | Gamma, Identity |
|  | High-density lipoprotein, HDL | mmol/L | Lipoprotein that binds cholesterol in circulation and carries to liver for disposal. Low values relate to increased risk of vascular disease | Kerr, 2002 | Gamma, Log |
|  | Low-density lipoprotein, LDL | mmol/L | Lipoprotein that binds circulating cholesterol, but via deposition of excess cholesterol in blood vessel walls. High values relate to increased risk of vascular disease | Kerr, 2002 | Gamma, Sqrt |
|  | Total cholesterol:HDL ratio, Ratio | - | Ratio used to evaluate lipid balance in organism | Kerr, 2002 | Gamma, Log |
| **Electrolytes** | Sodium,  Na | mmol/L | Electrolyte required for fluid retention, nerve, and muscle functions. Dependent on hydration status, body salt and water balances | Kerr, 2002 | Gamma, Log |
|  | Potassium,  K | mmol/L | Electrolyte required for cell metabolism, nerve stimulation, and muscle function | Kerr, 2002 | Gamma, Log |
|  | Chloride,  Cl | mmol/L | Electrolyte required for fluid retention and acid-base balance. Generally trends with Na except when acid-base imbalance occurs | Kerr, 2002 | Gamma, Identity |
| **Renal Function** | Urea,  Ur | mmol/L | Waste product produced in liver during protein breakdown, filtered from blood by kidneys. Can relate to liver and/or kidney functionality, along with protein consumption | Kerr, 2002 | Gamma, Identity |
|  | Creatinine,  Creat | umol/L | Waste product produced by muscle usage, filtered from blood by kidneys. Relates to kidney functions, particularly glomerular filtration rates | Kerr, 2002 | Gamma, Log |
|  | Uric acid,  UA | mmol/L | Waste produced in purine breakdown and end product of protein metabolism processes; removed from blood by kidneys. Relates to diet and kidney function | Kerr, 2002 | Binomial; Cloglog |
|  | Calcium,  Ca | mmol/L | Most prevalent mineral in body with multiple functions including muscular and cardiac processes, signaling, bone & teeth health, and blood clotting. Circulating levels strongly regulated by homeostatic feedback loops | Goltzman, 2018 | NA |
|  | Corrected calcium, Co_Ca | mmol/L | Second measure of calcium that accounts for protein- bound Ca; this reports 'free' & metabolically active circulating Ca. Preferred diagnostic | Goltzman, 2018 | Gamma, Identity |
|  | Phosphate,  P | mmol/L | Mineral required for muscle and nerve function, and bone and teeth development. Absorbed in intestines and excreted by kidneys. Alternative reporting name: Phosphorus | Kerr, 2002 | Gamma, Log |
| **Liver Function** | Total protein,  TotProt | g/L | Summary of circulating proteins in blood (equates to sum of albumin and globulins). Interpretation depends on albumin and immunoglobulin relations | Kerr, 2002 | Gamma, Identity |
|  | Albumin,  Alb | g/L | Protein produced by liver with multiple functions; responds to liver disease, hydration, and dietary status | Kerr, 2002 | Gamma, Log |
|  | Globulin,  Glo | g/L | Group of proteins (enzymes, antibodies, hormones, etc.) generated by liver and immune system; strongly related to inflammation | Kerr, 2002 | Gamma, Identity |
|  | Albumin:Globulin Ratio, AlbGloRatio | - | Generally >1 in humans. Can be indicator of recent disease exposure or system imbalances | Couch *et al*., 2017 | Gamma, Log |
|  | Alkaline phosphatase, AlkPho | U/L | Enzyme concentrated in liver and bone tissues. Elevation indicates diseases of these systems, although young organisms can have greater circulating concentrations due to skeletal growth. Alternative reporting name: ALP | Kerr, 2002 | Gamma, Log |
|  | Total bilirubin,  Bili | umol/L | Orange-yellow pigment that is the waste product of heme breakdown. Processed by the liver, and elevation indicates poor liver function | Kerr, 2002 | Binomial; Cloglog |
|  | Gamma-glutamyl transpeptidase,  GGT | U/L | Enzyme produced in liver with multiple functions; elevation indicates liver damage, particularly as relates to bile duct functionality and biliary disease. Quick response but non-specific; best diagnostically used in conjunction with ALP | Kerr, 2002 | Binomial; Cauchit |
|  | Aspartate aminotransferase,  AST | U/L | Enzyme produced in liver and muscle tissue with multiple functions. Circulatory levels routinely low, but will respond to liver or muscle damage (non-specific). Best diagnostically used in conjunction with ALT | Kerr, 2002 | Gamma, Log |
|  | Alanine transaminase, ALT | U/L | Enzyme found in liver and some kidney cells, it breaks down proteins. Circulatory levels routinely low, but will elevate following liver damage. More specific for hepatocellular damage than AST | Kerr, 2002 | Gamma, Log |

^*^Model family and link function refer to model structure of the statistical analyses conducted on each parameter; refer to data analysis section in the main text

**Supplementary Table 2:** Summary haematology profiles for wild Malay civets (*Viverra tangalunga*) sampled within the Lower Kinabatangan Floodplain from 2012-2019. Bold text denotes the directionality of significance, such that the bolded values represent the sex with the statistically greater values (*p*<0.05). Refer to Supplementary Table 1 for parameter shorthand reference. *Denotes significance for sex differences such that * *p*<0.05; ***p*<0.01. ^#^Indicates animal’s proximity to oil palm plantation was statistically significant in averaged GLM. @Indicates animal capture season was statistically significant in averaged GLM. ^&^Indicates individual’s age category was statistically significant in averaged GLM.

|  |  | **All civets** | | | |  | **Female civets** | | | |  | **Male civets** | | | |
| --- | --- | --- | --- | --- | --- | --- | --- | --- | --- | --- | --- | --- | --- | --- | --- |
| **Parameter** | **Unit** | **n** | **Mean (SD)** | **Median (IQR)** | **Min–Max** |  | **n** | **Mean (SD)** | **Median (IQR)** | **Min–Max** |  | **n** | **Mean (SD)** | **Median (IQR)** | **Min–Max** |
| Haem** | g/L | 51 | – | – | – |  | 26 | 102 (15.4) | 102 (20.5) | 73.0 – 131 |  | 25 | **120** (17.2) | 121 (15.0) | 73.0 – 149 |
| RBC**@ | x 10^12^ /L | 51 | – | – | – |  | 26 | 7.62 (1.28) | 7.60 (1.70) | 4.98 – 10.5 |  | 25 | **8.62** (1.26) | 8.85 (1.05) | 4.72 – 10.7 |
| PCV**@ | L/L | 51 | – | – | – |  | 26 | 0.40 (0.05) | 0.39 (0.08) | 0.30 – 0.50 |  | 25 | **0.46** (0.07) | 0.47 (0.11) | 0.31 – 0.58 |
| MCV | fL | 51 | 53.0 (4.57) | 53.0 (7.00) | 44.0 – 66.0 |  | – | – | – | – |  | – | – | – | – |
| MCH | pg | 51 | 13.7 (0.86) | 14.0 (1.0) | 11.0 – 16.0 |  | – | – | – | – |  | – | – | – | – |
| MCHC^#^ | g/L | 51 | 259 (16.8) | 256 (24.0) | 231 – 304 |  | – | – | – | – |  | – | – | – | – |
| RDW | % | 51 | 21.8 (1.99) | 21.6 (2.20) | 17.6 – 28.1 |  | – | – | – | – |  | – | – | – | – |
| WBC^#^ | x 10^9^/L | 51 | 11.5 (3.49) | 11.0 (4.35) | 5.70 – 23.3 |  | – | – | – | – |  | – | – | – | – |
| Neut^#^ | x 10^9^/L | 49 | 8.29 (3.36) | 7.40 (4.90) | 3.10 – 17.3 |  | – | – | – | – |  | – | – | – | – |
| Lymp*^&^ | x 10^9^/L | 49 | – | – | – |  | 25 | **2.81** (1.57) | 2.30 (1.40) | 0.60 – 6.90 |  | 24 | 1.98 (0.95) | 1.65 (1.30) | 0.60 – 4.20 |
| N:L | - | 49 | 4.8 (3.95) | 3.6 (3.43) | 0.78 – 17.8 |  | – | – | – | – |  | – | – | – | – |
| Mono | x 10^9^/L | 49 | 0.49 (0.39) | 0.40 (0.40) | 0.00 – 1.80 |  | – | – | – | – |  | – | – | – | – |
| Eos | x 10^9^/L | 31 | 0.42 (0.48) | 0.20 (0.60) | 0.00 – 1.80 |  | – | – | – | – |  | – | – | – | – |
| Baso | x 10^9^/L | 10 | 0.08 (0.22) | 0.00 (0.00) | 0.00 – 0.70 |  | – | – | – | – |  | – | – | – | – |
| Plat | x 10^9^/L | 50 | 307 (129) | 302 (142) | 88.0 – 677 |  | – | – | – | – |  | – | – | – | – |
| Gluc | mmol/L | 48 | 5.6 (1.5) | 5.7 (1.7) | 1.5 – 8.9 |  | – | – | – | – |  | – | – | – | – |

**Supplementary Table 3:** Summary serum biochemistry profiles for wild Malay civets (*Viverra tangalunga*) sampled within the Lower Kinabatangan Floodplain from 2012-2019. Bold text denotes the directionality of significance, such that the bolded values represent the age category with the statistically greater values (*p*<0.05). Refer to Supplementary Table 1 for parameter shorthand reference. *Denotes significance for age category differences such that * *p*<0.05; ***p*<0.01; ****p*<0.001. ^#^Indicates animal’s proximity to oil palm plantation was statistically significant in averaged GLM.

|  |  | **All civets** | | | |  | **Immature civets** | | | |  | **Mature civets** | | | |
| --- | --- | --- | --- | --- | --- | --- | --- | --- | --- | --- | --- | --- | --- | --- | --- |
| **Parameter** | **Unit** | **n** | **Mean (SD)** | **Median (IQR)** | **Min–Max** |  | **n** | **Mean (SD)** | **Median (IQR)** | **Min–Max** |  | **n** | **Mean (SD)** | **Median (IQR)** | **Min–Max** |
| Tchol | mmol/L | 56 | 3.4 (0.73) | 3.3 (0.73) | 2.5 – 5.6 |  | – | – | – | – |  | – | – | – | – |
| Tri | mmol/L | 56 | 0.74 (0.26) | 0.71 (032) | 0.37 – 1.67 |  | – | – | – | – |  | – | – | – | – |
| HDL^#^ | mmol/L | 56 | 2.16 (0.49) | 2.08 (0.75) | 1.39 – 3.25 |  | – | – | – | – |  | – | – | – | – |
| LDL | mmol/L | 56 | 0.916 (0.44) | 0.885 (0.56) | 0.03 – 2.02 |  | – | – | – | – |  | – | – | – | – |
| Ratio^#^ | - | 56 | 1.6 (0.21) | 1.6 (0.30) | 1.1 – 2.0 |  | – | – | – | – |  | – | – | – | – |
| Na | mmol/L | 56 | 150 (3.85) | 149 (5.00) | 141 – 159 |  | – | – | – | – |  | – | – | – | – |
| K | mmol/L | 39 | 4.2 (0.36) | 4.2 (0.50) | 3.5 – 5.0 |  | – | – | – | – |  | – | – | – | – |
| Cl | mmol/L | 56 | 118 (6.50) | 119 (8.50) | 103 – 131 |  | – | – | – | – |  | – | – | – | – |
| Ur^#^ | mmol/L | 55 | 8.2 (2.3) | 7.8 (2.6) | 3.30 – 14.2 |  | – | – | – | – |  | – | – | – | – |
| Creat* | umol/L | – | – | – | – |  | 13 | 58 (12) | 58 (10) | 37 – 81 |  | 42 | **68** (13) | 68 (19) | 40 – 90 |
| Co_Ca | mmol/L | 38 | 2.34 (0.14) | 2.32 (0.15) | 2.03 – 2.78 |  | – | – | – | – |  | – | – | – | – |
| P*** | mmol/L | – | – | – | – |  | 13 | **1.93** (0.69) | 1.87 (0.55) | 0.99 – 3.68 |  | 43 | 1.46 (0.30) | 1.43 (0.31) | 0.92 – 2.13 |
| TotProt** | g/L | – | – | – | – |  | 13 | 74.8 (8.88) | 77.0 (16.0) | 61.0 – 86.0 |  | 43 | **81.0** (6.30) | 82.0 (9.50) | 67.0 – 92.0 |
| Alb | g/L | 53 | 28.9 (4.22) | 29.0 (4.00) | 10.0 – 37.0 |  | – | – | – | – |  | – | – | – | – |
| Glob*** | g/L | – | – | – | – |  | 13 | 46.1 (8.11) | 45.0 (11.0) | 32.0 – 59.0 |  | 40 | **52.2** (6.83) | 52.0 (6.00) | 39.0 – 82.0 |
| AlbGloRatio | - | 53 | 0.58 (0.13) | 0.60 (0.16) | 0.10 – 0.90 |  | – | – | – | – |  | – | – | – | – |
| AlkPho* | U/L | – | – | – | – |  | 7 | **57.4** (33.3) | 52.0 (14.5) | 22.0 – 127 |  | 28 | 27.0 (19.2) | 17.5 (23.5) | 7.0 – 71 |
| AST | U/L | 56 | 183.0 (108.0) | 148.0 (102.0) | 17.0 – 559 |  | – | – | – | – |  | – | – | – | – |
| ALT | U/L | 54 | 152.0 (84.0) | 130.0 (67.8) | 48.0 – 448 |  | – | – | – | – |  | – | – | – | – |

**Supplementary Table 4:** Select summary serum biochemistry profiles for wild Malay civets (*Viverra tangalunga*) sampled within the Lower Kinabatangan Floodplain from 2012-2019. Descriptive statistics are calculated only from those individuals with values over the laboratory’s reported limit of detection (L.D.). Refer to Supplementary Table 1 for parameter shorthand reference.

| **Parameter** | **L.D.** | **n < L.D.** | **n Elevated** | **Mean (SD)** | **Median (IQR)** | **Min ­– Max** |
| --- | --- | --- | --- | --- | --- | --- |
| UA | 0.01 mmol/L | 44 | 11 | 0.011 (0.0030) | 0.01 (0.00) | 0.01 – 0.02 |
| Bili | 2 umol/L | 36 | 19 | 16.6 (58.0) | 3.0 (2.0) | 2 – 256 |
| GGT | 3 U/L | 18 | 37 | 20.5 (33.1) | 9.0 (14.0) | 3 – 195 |

**Supplementary Table 5:** Select biochemistry parameters of GPS collared male Malay civets (*Viverra tangalunga*) with home ranges solely in the forest and those known to enter oil palm plantations. Descriptive statistics are calculated only from those individuals with values over the laboratory’s reported limit of detection (L.D.). Refer to Supplementary Table 1 for parameter shorthand reference.

|  |  | **Forest Only** | | | | |  | **Mix** | | | | |
| --- | --- | --- | --- | --- | --- | --- | --- | --- | --- | --- | --- | --- |
| **Parameter** | **L.D.** | **n < L.D.** | **n Elevated** | **Mean (SD)** | **Median (IQR)** | **Min – Max** |  | **n < L.D.** | **n Elevated** | **Mean (SD)** | **Median (IQR)** | **Min – Max** |
| UA | 0.01 mmol/L | 4 | 1 | 0.05 (NA) | 0.05 (NA) | NA |  | 6 | 2 | 0.01 (0.0) | 0.01 (0.0) | 0.01 – 0.01 |
| Bili | 2 umol/L | 2 | 3 | 3.3 (1.5) | 3.0 (1.5) | 2 – 5 |  | 3 | 5 | 4.0 (2.9) | 3.0 (2.0) | 2 – 9 |
| GGT | 3 U/L | 1 | 4 | 29 (22.8) | 27.5 (20.5) | 3 – 58 |  | 3 | 5 | 46.2 (83.3) | 9.0 (9.0) | 6 – 195 |

**Supplementary Table 6:** Summary blood parameters of GPS collared male Malay civets (*Viverra tangalunga*) with home ranges solely in the forest and those known to enter oil palm plantations. Bold text denotes the directionality of significance, such that bolded values represent the dataset with statistically elevated parameters. Parameter shorthand matches those presented in Supplementary Table 1. *denotes statistical significance: * *p* value < 0.05; ** *p* value < 0.01; ‘ *p* value < 0.08.

| **Parameter** | **Unit** | **Forest Only** | | |  | **Mix** | | |
| --- | --- | --- | --- | --- | --- | --- | --- | --- |
|  |  | **n** | **Mean (SD)** | **Median (IQR)** |  | **n** | **Mean (SD)** | **Median (IQR)** |
| Haem | g/L | 5 | 124 (7.84) | 126 (8.00) |  | 8 | 118 (17.5) | 120 (21.2) |
| RBC | x 10^12^/L | 5 | 8.83 (0.91) | 8.96 (1.05) |  | 8 | 8.58 (1.17) | 8.58 (1.37) |
| PCV | L/L | 5 | 0.45 (0.049) | 0.47 (0.090) |  | 8 | 0.47 (0.069) | 0.48 (0.113) |
| MCV* | fL | 5 | 50.8 (2.7) | 51.0 (2.0) |  | 8 | **54.9** (3.4) | 53.5 (3.5) |
| MCH | pg | 5 | 14.2 (1.1) | 14.0 (0.0) |  | 8 | 13.9 (0.6) | 14 (0.3) |
| MCHC** | g/L | 5 | **278** (18.3) | 276 (19.0) |  | 8 | 251 (12.6) | 250 (16.8) |
| RDW | % | 5 | 21.2 (1.12) | 21.4 (1.70) |  | 8 | 22.3 (3.34) | 21.5 (3.15) |
| WBC | x 10^9^/L | 5 | 13.6 (5.86) | 11.5 (4.20) |  | 8 | 10.0 (2.12) | 9.60 (2.35) |
| Neut | x 10^9^/L | 5 | 10.10 (4.69) | 8.60 (6.10) |  | 7 | 7.90 (2.55) | 6.40 (4.70) |
| Lymp | x 10^9^/L | 5 | 2.24 (1.21) | 1.70 (1.20) |  | 7 | 1.80 (1.16) | 1.60 (1.90) |
| N:L | - | 5 | 5.0 (2.49) | 4.15 (1.06) |  | 7 | 7.5 (6.94) | 3.62 (8.70) |
| Mono’ | x 10^9^/L | 5 | 0.76 (0.49) | 0.70 (0.80) |  | 7 | 0.33 (0.33) | 0.20 (0.30) |
| Plat | x 10^9^/L | 5 | 231 (78.8) | 249 (149) |  | 8 | 358 (154) | 340 (89.0) |
| Gluc | mmol/L | 5 | 5.7 (2.7) | 5.8 (3.0) |  | 8 | 5.6 (1.98) | 5.5 (2.37) |
| Tchol | mmol/L | 5 | 3.3 (0.48) | 3.3 (0.50) |  | 8 | 3.4 (0.75) | 3.3 (0.35) |
| Tri | mmol/L | 5 | 0.71 (0.23) | 0.70 (0.23) |  | 8 | 0.74 (0.23) | 0.69 (0.31) |
| HDL | mmol/L | 5 | 1.92 (0.36) | 1.93 (0.41) |  | 8 | 2.18 (0.47) | 2.08 (0.57) |
| LDL | mmol/L | 5 | 1.22 (0.30) | 1.32 (0.49) |  | 8 | 0.87 (0.58) | 0.84 (0.48) |
| Ratio | - | 5 | 1.7 (0.21) | 1.7 (0.30) |  | 8 | 1.6 (0.25) | 1.6 (0.25) |
| Na | mmol/L | 5 | 148 (3.21) | 148 (4.00) |  | 8 | 150 (4.85) | 150 (5.25) |
| K | mmol/L | 5 | 4.2 (0.45) | 4.1 (0.30) |  | 6 | 4.2 (0.28) | 4.2 (0.18) |
| Cl | mmol/L | 5 | 118 (3.97) | 117 (2.00) |  | 8 | 116 (7.61) | 118 (11.0) |
| Ur** | mmol/L | 5 | **11.7** (1.47) | 11.9 (1.20) |  | 8 | 7.69 (1.66) | 7.8 (2.28) |
| Creat | umol/L | 5 | 70.4 (16.1) | 75.0 (5.0) |  | 8 | 72.4 (13.2) | 68.5 (22.2) |
| Co_Ca | mmol/L | 5 | 2.37 (0.11) | 2.38 (0.12) |  | 6 | 2.45 (0.19) | 2.40 (0.21) |
| P | mmol/L | 5 | 1.59 (0.53) | 1.34 (0.36) |  | 8 | 1.64 (0.41) | 1.78 (0.65) |
| TotProt | g/L | 5 | 82.6 (8.35) | 82.0 (4.00) |  | 8 | 78.5 (7.01) | 81.5 (9.75) |
| Alb | g/L | 5 | 30.8 (1.92) | 30.0 (1.00) |  | 8 | 27.1 (8.53) | 28.5 (4.50) |
| Glob | g/L | 5 | 51.8 (7.79) | 52.0 (1.00) |  | 8 | 51.4 (8.02) | 52.0 (6.50) |
| AlbGloRatio | - | 5 | 0.62 (0.08) | 0.6 (0.10) |  | 8 | 0.55 (0.19) | 0.6 (0.20) |
| AlkPho | U/L | 5 | 51.2 (44.0) | 45.0 (42.0) |  | 6 | 19.9 (12.3) | 19.0 (3.5) |
| AST | U/L | 5 | 262 (174) | 215 (109) |  | 8 | 236 (153) | 220 (259) |
| ALT | U/L | 5 | 99.2 (33.4) | 106 (40.0) |  | 8 | 176.0 (129.0) | 154 (82.2) |
